# How Should Bacteriological Sampling Be Stratified in Paediatric Septic Arthritis? A Narrative Review with a Proposed Risk-Stratified Framework

**DOI:** 10.3390/antibiotics15080719

**Published:** 2026-07-24

**Authors:** Pablo Rodriguez, Maxime Schilliger, Ahmer Khan, Giacomo De Marco, Oscar Vazquez, Andreas Tsoupras, Ardian Ramadani, Christina Steiger, Romain Dayer, Dimitri Ceroni

**Affiliations:** 1 Orthopaedics and Traumatology Unit, Surgery Department, Geneva University Hospitals, CH-1205 Geneva, Switzerland; 2 Paediatric Orthopaedics Unit, Service of Paediatric Surgery, Department of Women, Children and Adolescents, Geneva University Hospitals, CH-1205 Geneva, Switzerland

**Keywords:** paediatric, septic arthritis, joint aspiration, bacteriological sampling, toxins, MRSA, *Staphylococcus aureus*, *Kingella kingae*, risk stratification, narrative review

## Abstract

Joint aspiration remains the gold standard and an urgent step in the diagnosis of paediatric septic arthritis (SA). Unlike in acute haematogenous osteomyelitis, it has both diagnostic and therapeutic value. No previous review has specifically addressed when bacteriological sampling is essential and when it may reasonably be omitted. The bacteriological profile in children is highly age-dependent: *Kingella kingae* predominates before 4 years of age, whereas *Staphylococcus aureus*—including Panton–Valentine leukocidin (PVL)-producing and methicillin-resistant (MRSA) strains—predominates thereafter. We critically review the evidence through nine clinical questions and propose a conceptual risk-stratified framework in which the sampling approach is tailored to age and clinical context. In children younger than 4 years with a positive oropharyngeal *K. kingae* PCR and a mild clinical presentation—defined as CRP < 20 mg/L, absence of fever, and preserved weight-bearing—non-invasive confirmation may be sufficient. This proposal is explicitly hypothesis-generating: it is derived from observational data, it has not been validated prospectively, and it is not endorsed by current PIDS/IDSA or ESPID guidance. A positive oropharyngeal PCR alone is never sufficient, given the 10–12% asymptomatic carriage rate and the limited reliability of the Kocher–Caird criteria in this age group; the decision requires a cluster of concordant findings together with mandatory clinical and laboratory reassessment at 48–72 h and a low threshold for escalation to arthrocentesis. In children older than 4 years, arthrocentesis under general anaesthesia remains the standard approach, with pathogen identification and antimicrobial susceptibility testing as the primary microbiological determinants of therapy and toxin profiling as an adjunctive investigation in selected cases.

## 1. Introduction

Paediatric septic arthritis (SA) is a severe infection caused by bacterial invasion of the joint space, with the potential for rapid cartilage destruction and serious long-term sequelae if treatment is delayed [1,2,3]. In large European series, SA represents about 40–45% of paediatric osteoarticular infections (OAIs), most commonly affecting the hip, knee and ankle, in that order [3]. In developed countries, its annual incidence is estimated at 4–5 cases per 100,000 children, with a male predominance and a bimodal age distribution, peaking in early childhood (6 months to 4 years) and again during adolescence (12–16 years) [3,4].

Joint aspiration has a different role in SA than bone biopsy does in acute haematogenous osteomyelitis (AHO). In AHO without abscess, invasive sampling is primarily diagnostic: bone aspirate or biopsy may identify the pathogen, but it provides no therapeutic benefit in uncomplicated cases, especially when fastidious organisms are involved. In SA, by contrast, joint aspiration is both diagnostic and therapeutic [1,2,5]. By obtaining fluid for microbiological analysis, it also decompresses the joint, lowers intra-articular pressure, and helps limit cartilage damage caused by both effusion and the proteolytic effects of purulent fluid [1,3]. This dual function creates a clinical continuum specific to SA: suspicion leads to aspiration; purulent fluid may then prompt immediate surgical lavage—by syringe, arthroscopy, or arthrotomy—and culture results may subsequently identify a virulent organism and guide further management [2,6].

The pathophysiology of joint damage in SA involves also a rapid and destructive inflammatory cascade. When the infection is not quickly controlled, the immune response generates high levels of cytokines and reactive oxygen species that stimulate the release of host matrix metalloproteinases and other collagen-degrading enzymes; together with bacterial toxins, these mediators cause progressive joint destruction [7,8]. Experimental studies have shown that degradation of articular cartilage begins within hours of infection, and that irreversible destruction of the intra-articular cartilage with subchondral bone loss can occur in as little as three days [7,9]. Importantly, metalloproteinases and the antigen-driven inflammatory response may persist and continue to erode the joint architecture even after the infection has been eradicated [10,11]. This pathophysiological cascade provides a strong rationale for both prompt joint lavage—to remove the proteolytic environment—and early pathogen-directed therapy.

Certain bacterial virulence factors—particularly exotoxins such as Panton–Valentine leukocidin (PVL) produced by some *Staphylococcus aureus* strains—are associated with markedly more severe disease, including deep vein thrombosis, septic emboli, necrotising pneumonia and multifocal infection [12,13,14,15]. Identifying these factors through bacteriological sampling may therefore alter management, including the use of anti-toxin therapy (clindamycin, linezolid), the need for repeated surgical lavage, and the overall intensity of medical and surgical treatment [16,17]. Accordingly, the clinical question has shifted from ‘should we sample?’ to ‘what additional information can sampling provide to optimise treatment?’

Despite the clinical relevance of these issues, no narrative review has specifically focused on the indications for bacteriological sampling in paediatric SA. Previous reviews have considered OAIs more broadly [3,18] or examined specific topics such as culture methods [19], molecular diagnostics [20,21], or clinical management [4,6]. To address this gap, we critically appraised the evidence specific to SA and integrated emerging data on virulence factors into a risk-stratified sampling framework. Our aim is therefore to define when and how bacteriological sampling should be performed, and to propose a risk-adapted framework: comprehensive sampling with toxin profiling when a virulent organism is suspected, and a less invasive strategy in young children with mild, low-risk presentations. The review is organised around nine clinical questions.

## 2. Methodology

This narrative review critically appraised the evidence guiding bacteriological sampling in paediatric SA, examined how pathogen virulence and age-related epidemiology influence sampling decisions, and proposed an SA-specific risk-stratified framework. Relevant studies were identified through a structured literature search.

Following the recommendations of Pai et al. [22] and with support from an experienced research librarian, we developed Boolean search strategies for PubMed/MEDLINE, Embase, and the Cochrane Library. The search covered publications from January 1997 to March 2026 and was limited to English- and French-language original studies, systematic reviews, clinical guidelines, narrative reviews, and case series. Core search terms included paediatric, septic arthritis, joint aspiration, arthrocentesis, bacteriological sampling, blood culture, culture-negative, PCR, Panton–Valentine leukocidin, PVL, virulence factors, and exotoxin. Additional section-specific terms included blood culture bottle inoculation, metagenomic next-generation sequencing, antibiotic pretreatment, clinical prediction, Kocher criteria, bedside aspiration, and anti-toxin therapy. We excluded studies limited to adults, implant- or prosthesis-related infections, case reports, and articles that did not address sampling indications or virulence factors.

We specifically sought guidelines from the Pediatric Infectious Diseases Society and the Infectious Diseases Society of America (PIDS/IDSA; 2021 for AHO [1], 2023 for acute bacterial arthritis [2]) and from the European Society for Paediatric Infectious Diseases (ESPID; 2017 [3]), as well as national guidance from Health Protection Agency (HPA) and the Groupe de Pathologie Infectieuse Pédiatrique (GPIP). The 2021 PIDS/IDSA AHO guideline was included because several recommendations on culture techniques and antibiotic pretreatment are also relevant to SA sampling. We also hand-searched the reference lists of included articles for additional studies. All references were checked against the original source documents, and author names, journal titles, publication years, and DOIs (when available) were verified for accuracy.

The search identified 299 publications. After abstract screening, 91 were excluded, leaving 208 articles for full-text review; a total of 88 were ultimately retained and cited. In response to peer review, a further targeted search on biochemical markers in paediatric SA identified three additional publications, giving 91 references in total. Because this is a narrative review, the selection process did not follow a formal PRISMA-compliant protocol. This was a deliberate choice, given the heterogeneity of study designs, outcomes, and clinical settings across the nine clinical questions, which precluded meaningful quantitative synthesis. The evidence base consists mainly of retrospective observational studies and case series, and no randomised controlled trial has evaluated sampling strategies in paediatric SA. Where available, we preferentially cited meta-analyses, systematic reviews, and prospective cohort studies to ground the discussion in the highest level of evidence available.

## 3. From Evidence to a Risk-Stratified Sampling Framework

The following sections examine the evidence through nine clinical questions that together inform a risk-stratified approach to bacteriological sampling in paediatric SA. We first outline the epidemiological and microbiological background, then show how these data guide the organisation of sampling and the selection of diagnostic methods. Appendix D summarises the key studies underpinning this framework.

### 3.1. How Does Age-Related Bacteriological Epidemiology Shape the Sampling Strategy?

**Figure 1:**
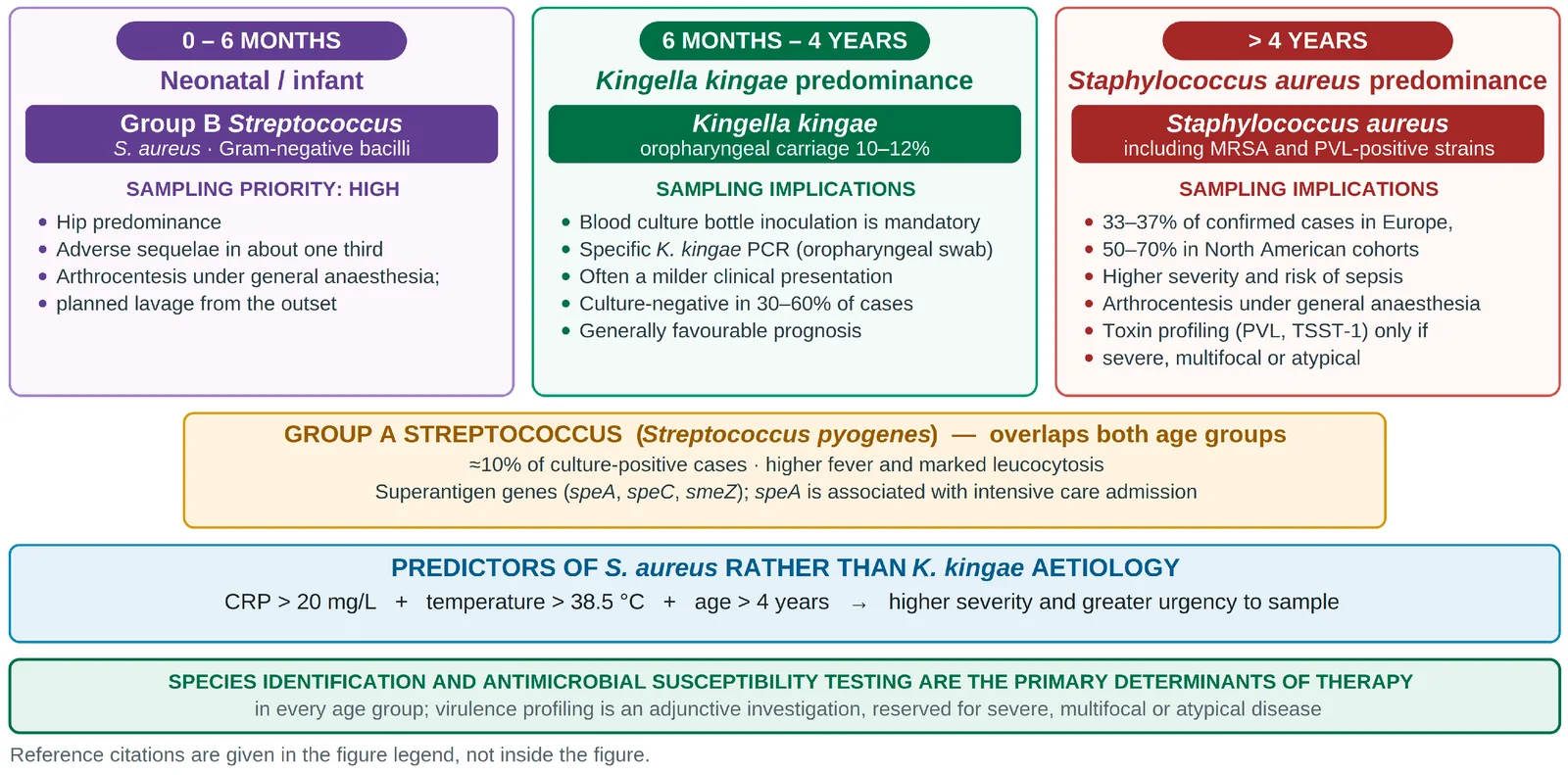
Age-dependent pathogen distribution and its implications for bacteriological sampling in paediatric septic arthritis. In neonates and young infants, group B *Streptococcus*, *S. aureus* and Gram-negative bacilli predominate; the hip is most often involved and about one third of neonates develop adverse sequelae, so arthrocentesis under general anaesthesia with planned lavage is recommended [38,39,40]. Between 6 months and 4 years, *Kingella kingae* predominates, with an asymptomatic oropharyngeal carriage rate of 10–12% [30]; conventional culture is negative in 30–60% of cases, which makes inoculation into blood culture bottles and specific *K. kingae* PCR essential [1,19,20,21,25], while the disease usually follows a milder course [26] with a favourable prognosis [41]. Beyond 4 years, *Staphylococcus aureus* predominates, accounting for 33–37% of confirmed cases in European series [3,23,24] and 50–70% in North American cohorts, where community-associated MRSA is more frequent [1,33,34]; severity and the risk of sepsis are higher, arthrocentesis under general anaesthesia is standard, and toxin profiling (PVL, TSST-1) is reserved for severe, multifocal or atypical disease [16,17]. Group A *Streptococcus* overlaps both age groups, accounts for roughly 10% of culture-positive cases [36], and carries superantigen genes (*speA*, *speC*, *smeZ*), of which *speA* is associated with intensive care admission [42]. The predictors of an *S. aureus* rather than a *K. kingae* aetiology are a CRP > 20 mg/L, a temperature >38.5 °C and an age over 4 years [43,44]. In every age group, species identification and antimicrobial susceptibility testing remain the primary determinants of therapy, and virulence profiling is an adjunctive investigation [1,2]. For legibility, reference citations are given in this legend rather than within the figure. CRP = C-reactive protein; MRSA = methicillin-resistant *Staphylococcus aureus*; PCR = polymerase chain reaction; PVL = Panton–Valentine leukocidin; *speA*, *speC*, *smeZ* = streptococcal superantigen-encoding genes; TSST-1 = toxic shock syndrome toxin-1.

The bacteriology of paediatric SA is strongly age-dependent, and this epidemiological pattern directly shapes the sampling strategy (Figure 1). In Western Europe, current evidence indicates that about 90% of primary paediatric OAIs are caused by three pathogen groups: *Kingella kingae*, *Staphylococcus aureus*, and streptococcal species [3,23,24].

First, *K. kingae* is the leading pathogen in children younger than 4 years and accounts for most microbiologically confirmed OAIs in European series [20,24,25,26,27,28,29]. These infections usually present mildly: fewer than 15% of patients are febrile, and about 40% have normal CRP levels [26]. *K. kingae* is an oropharyngeal commensal in young children, with carriage rates peaking at 10–12% among daycare attendees aged 6–48 months [30]. It is also difficult to detect by conventional culture methods, as direct Gram stain and standard culture are often negative in cases later identified by specific PCR [19,25,26]. Oropharyngeal swab PCR can therefore serve as a non-invasive diagnostic adjunct: in a child with clinical features of OAI, a positive result may support the diagnosis without invasive joint aspiration [31]. This approach has also been validated in spondylodiscitis [32]. Although oropharyngeal PCR cannot replace joint fluid analysis in every situation—particularly when joint decompression or surgical lavage is clinically indicated—it offers a less invasive first-line diagnostic option. In young children with mild symptoms and a positive oropharyngeal PCR, whether joint aspiration is routinely required remains an open question and should be viewed as a conceptual proposal pending prospective validation (see Section 4.1).

In children older than 4 years, *S. aureus* is the predominant pathogen, accounting for 33–37% of confirmed cases in European series [3,23,24] and 50–70% in North American cohorts, where community-associated methicillin-resistant *S. aureus* (CA-MRSA) is more frequent [1,33,34]. In Europe, MRSA prevalence is generally low: in the EUCLIDS study, only 0.7% of 141 culture-confirmed staphylococcal OAIs were caused by MRSA [35]. Empirical narrow-spectrum anti-staphylococcal therapy is therefore often appropriate.

Among streptococci, *Streptococcus pyogenes* accounts for roughly 10% of culture-positive cases and is associated with more severe presentations, including higher fever and marked leucocytosis [36]. In contrast, *Streptococcus pneumoniae*, once a frequent cause of OAI in young children, has become far less common since the introduction of pneumococcal conjugate vaccines [37].

This age-dependent bacteriological pattern directly determines the sampling strategy. In younger children, where *K. kingae* predominates and conventional culture is often unhelpful, microbiological confirmation should rely mainly on molecular methods, including specific PCR on joint fluid or oropharyngeal swab PCR [23]. In older children, where *S. aureus* predominates, joint fluid culture remains more informative, particularly when susceptibility testing or virulence factor analysis may affect management.

### 3.2. How Do Virulent Organisms and Their Toxins Influence the Sampling Decision?

This section focuses on *S. aureus*, the second most common cause of paediatric SA. Its pathogenicity is driven by a broad array of virulence factors, including toxins (PVL, TSST-1), enzymes (proteases, lipases, nucleases, hyaluronidase, staphylokinase), and surface proteins (ClfA, ClfB, and fibronectin-binding proteins); all these elements promote host colonisation, immune evasion, and severe infection. In addition, the accessory gene regulator (*agr*) system is a key controller of *S. aureus* virulence and its allelic group can now be characterised.

#### 3.2.1. Panton–Valentine Leukocidin (PVL) as Example of Exotoxin

PVL is a two-component pore-forming toxin encoded by the *lukS-PV* and *lukF-PV* genes. It destroys human neutrophils, thereby weakening the host’s primary defence against staphylococcal infection [45,46]. PVL also promotes a thrombotic cascade, which likely contributes to the deep vein thromboses and septic pulmonary emboli observed in paediatric PVL-positive OAIs [12,15,47].

Experimental and clinical data consistently associate PVL with more severe disease. In a rabbit model, Crémieux et al. showed that PVL-positive CA-MRSA caused more severe osteomyelitis than an isogenic PVL-deleted mutant, with persistent infection at day 28 in 100% versus 0% of cases [48]. Paediatric cohorts support these findings and further indicate an association with multifocal and disseminated infection. Bocchini et al. reported that children with PVL-positive AHO had higher CRP levels, more frequent bacteraemia (67.2% vs. 19.2%), and more ICU admissions (18.6% vs. 3.8%) [12]. In the European reference study by Dohin et al. including 14 PVL-positive and 17 PVL-negative cases, severity was even more marked, with septic shock in 43% versus 0%, surgical intervention in 71% versus 18%, and hospital stays of 45 versus 13 days [13]. In a cohort of 131 paediatric OAIs, Cui et al. identified CRP > 162 mg/L, delayed surgical intervention >9.5 days, bacteraemia, and ≥2 operations as independent risk factors for dissemination [49].

However, the link between PVL and orthopaedic complications is less clear. In the largest single-centre cohort (286 paediatric OAIs), McNeil et al. found no significant association between PVL positivity and orthopaedic complications [50]. PVL therefore appears to be a marker of systemic severity—such as septic shock, ICU admission, and disseminated disease—rather than a direct predictor of orthopaedic sequelae. By contrast, the *agr* (accessory gene regulator) group III genotype, a regulatory system controlling the expression of multiple *S. aureus* virulence factors, was independently associated with chronic osteomyelitis (27.2% vs. 8.9%). Finally, delayed source control, fever persisting beyond 4 days, and ICU admission may be stronger predictors of complications [50].

Overall, orthopaedic sequelae seem to depend more on regulatory genotypes such as *agr* group III and on the timeliness of surgical management than on PVL alone, underscoring the interplay between bacterial virulence, host response, and adequacy of source control. However, most of these data come from osteomyelitis or mixed osteoarticular infections rather than from isolated septic arthritis [12,13,49,50].

#### 3.2.2. Regional Epidemiology of PVL and MRSA

The prevalence of PVL-positive *S. aureus* and MRSA varies widely by region and may affect sampling strategy. In the EUCLIDS cohort (see Section 3.1), 4.2% of culture-confirmed staphylococcal OAIs were PVL-positive [35]. In Switzerland and other Western European countries, PVL and MRSA remain uncommon but are not absent. By contrast, much higher rates have been reported in Greece and North America. Bouras et al. (123 children) found MRSA in 35.8% of cases, with PVL positivity in 92.3% of MRSA isolates versus 4.8% of MSSA isolates [51]. In Belgium, Goemanne et al. described severe PVL-positive MSSA infections in children of African origin and reported PVL positivity in 21.6% of community MSSA isolates [52]. This higher prevalence of PVL-positive MSSA in African settings is supported by Moutaouakkil et al., who reported an overall PVL positivity rate of 46% in Morocco [53]. A global meta-analysis by Al-Iede et al. (124 studies; 44 million children) estimated MRSA colonisation prevalence at 8% in Asia, 5% in the Americas, and 3% in Europe [54]. Aguilar-Gómez et al. further illustrated this geographic variability by reporting multidrug resistance in 78% of *S. aureus* isolates from paediatric OAIs in Mexico City [34]. Increased vigilance is therefore warranted in populations with epidemiological links to regions where these strains are more prevalent [35,52,55].

#### 3.2.3. Detection of Virulence Factors: Available Methods

For *S. aureus*, three complementary methods can be used to detect PVL. PCR remains the reference standard, with sensitivities of 95–100% and specificities close to 100% compared with culture-based methods [56]. Renwick et al. developed a multiplex real-time PCR that simultaneously detects MRSA and PVL directly from clinical specimens, with a detection threshold of 100 CFU per reaction [56]. ELISA provides direct PVL protein detection with 90% sensitivity and 100% specificity [57]. An immunochromatographic test (ICT) offers a rapid point-of-care alternative, yielding results within 30 min with 79% sensitivity and 100% specificity [57]. Because all three methods are highly specific, a positive result strongly supports PVL production. By contrast, the lower sensitivity of ICT means that negative point-of-care results should be confirmed by PCR when clinical suspicion remains high.

TSST-1 (toxic shock syndrome toxin-1) is another relevant virulence factor and can be detected either by PCR targeting the *tst* gene or by ELISA on bacterial isolates [58]. Whenever a staphylococcal isolate is available, comprehensive toxin profiling should therefore include both PVL and TSST-1.

In *Streptococcus pyogenes*, superantigen genes—particularly *speA*, which is associated with paediatric intensive care unit admission—can be detected by PCR on cultured isolates, with high sensitivity once the organism has recovered [42]. During the 2022–2023 post-pandemic European surge in invasive Group A *Streptococcus* infection, Ramírez de Arellano et al. found osteoarticular involvement in 11.8% of 102 paediatric cases, with *emm1* and *emm12* as the predominant genotypes [42].

#### 3.2.4. How Virulence Factor Identification Influences Treatment

Identifying virulence factors may have immediate therapeutic consequences. In PVL-positive infections, anti-toxin agents such as clindamycin or linezolid are strongly recommended in addition to standard anti-staphylococcal therapy because they inhibit ribosomal protein synthesis and thereby reduce toxin production [16].

The CASSETTE trial (Campbell et al.), the only controlled study to assess adjunctive clindamycin in severe *S. aureus* osteoarticular infections, randomised 34 patients (17 per group, including 11 children) [59]. Although the primary endpoint—days alive without systemic inflammatory response syndrome (SIRS)—was not statistically significant, 90-day mortality was 0% in the clindamycin group versus 24% with standard care. This clinically relevant difference did not reach statistical significance because the trial was stopped early after enrolling only 34 of the 60 planned participants [59]. Given the small sample size and premature termination, these findings should be viewed as hypothesis-generating rather than definitive evidence for adjunctive anti-toxin therapy.

These findings show that bacteriological sampling in SA serves not only to identify the pathogen and guide antibiotic choice but also to detect virulence factors that may influence the intensity of both medical and surgical treatment. This provides an additional rationale for sampling beyond pathogen identification alone.

### 3.3. How Does the Diagnostic–Therapeutic Continuum of Joint Aspiration Shape the Indication for Sampling?

In SA, joint aspirations have both diagnostic and therapeutic value, creating a clinical continuum that shapes its practical use [1,2,5]. This continuum can be summarised in six steps: (1) clinical suspicion based on symptoms, inflammatory markers, and imaging; (2) indication for joint aspiration, usually under general anaesthesia in young children; (3) arthrocentesis with macroscopic assessment of the aspirated fluid; (4) if the fluid is purulent, immediate joint lavage—by syringe, arthroscopy, or arthrotomy—during the same anaesthetic session; (5) microbiological analysis, including culture, antibiogram, and, when appropriate, toxin profiling (PVL, TSST-1); and (6) if a virulent organism is identified, adjustment of antibiotic therapy and consideration of repeat surgical lavage [2,6,16].

In many paediatric centres in Europe, arthrocentesis under general anaesthesia in the operating room remains standard practice. This allows immediate progression to joint lavage during the same anaesthetic session if the aspirated fluid is purulent [1,2,6]. The approach offers four main advantages: optimal aseptic conditions, a single anaesthetic event combining diagnosis and treatment, avoidance of repeated anaesthetic exposure in young children, and the option to escalate promptly to arthroscopy or arthrotomy if needed. In this setting, the transition from aspiration to surgical lavage is seamless, with the decision guided intraoperatively by the macroscopic appearance of the aspirate.

An alternative strategy has emerged in several North American centres, where arthrocentesis is performed under procedural sedation outside the operating room—commonly termed ‘bedside aspiration’. In a series of 62 patients, Skaggs et al. reported that this approach reduced the time to joint fluid collection from 15.7 h with operating room aspiration to 7.4 h with bedside aspiration [60]. In that cohort, 54% of children who underwent bedside aspiration did not subsequently require irrigation and debridement [60]. Braig et al. reported similar results in a multicentre study of 233 patients (75 operating room versus 158 bedside aspirations), in which 49% of bedside-aspirated children avoided both operating room intervention and general anaesthesia [61]. Thus, only about half of the children ultimately required a second anaesthetic event for operative lavage. Although aseptic conditions outside the operating room have not been formally compared with those in the operative setting, this approach may be particularly relevant where timely access to the operating room is limited. In this context, a survey of 18 US tertiary centres confirmed substantial practice variation, with hip aspirations evenly split between general anaesthesia and procedural sedation [5].

The suspected pathogen also plays a central role in determining the indication for diagnostic and therapeutic joint aspiration. In children younger than 4 years, oropharyngeal swab PCR for *K. kingae* is a key decision point that may help distinguish between non-invasive confirmation and arthrocentesis [31,32]. In children older than 4 years, arthrocentesis under general anaesthesia with culture, antibiogram, and toxin profiling remains the standard [1,2,16,17]. In severely ill children, regardless of age, joint lavage is planned from the outset as part of a combined diagnostic–therapeutic procedure [2,6,16]. As said just above, bedside aspiration may be an emerging alternative [60,61], but about half of children would probably require a second anaesthetic event for operative lavage, reserving this attitude for situations with limited timely access to the operating room.

**Figure 2:**
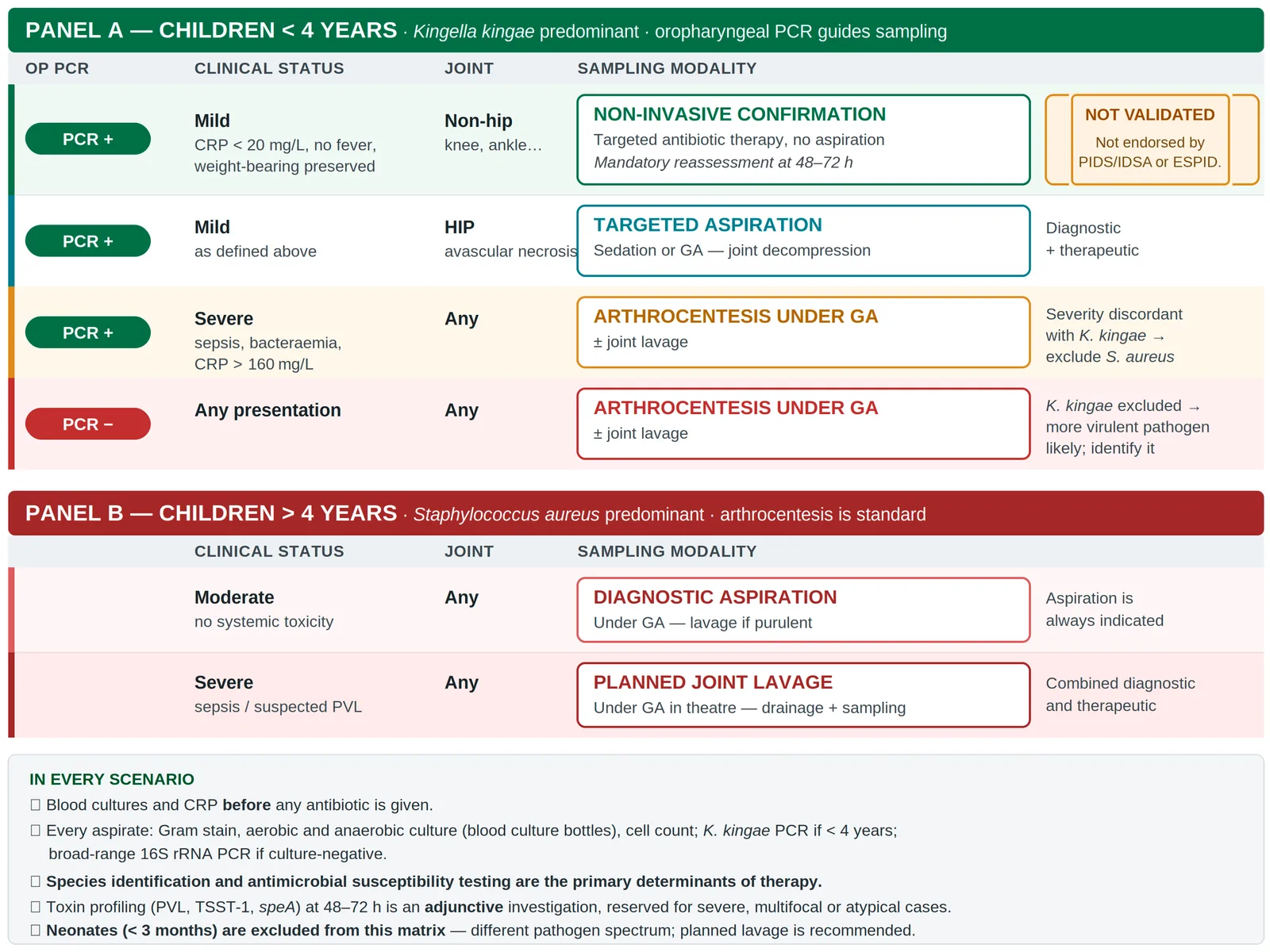
Decision matrix for bacteriological sampling modality in paediatric septic arthritis, stratified by age, oropharyngeal *K. kingae* PCR result, clinical severity and joint site. Panel (**A**): children < 4 years (four scenarios guided by the OP PCR result, clinical severity and joint site). Panel (**B**): children > 4 years (*S. aureus* predominant; arthrocentesis under GA is standard). A mild presentation requires all three of the following: CRP < 20 mg/L, absence of fever (<38.5 °C) and preserved weight-bearing or preserved active use of the limb (see Section 4.1). Universal prerequisites: blood cultures and CRP before antibiotics [2]. Every aspirate undergoes culture, species identification and antimicrobial susceptibility testing, which are the primary microbiological determinants of therapy [1,2]; toxin profiling at 48–72 h is an adjunctive investigation reserved for severe, multifocal or atypical cases [16,17,42]. Neonates are excluded (different pathogen spectrum; planned lavage recommended [38,39,40]). The non-invasive pathway (Panel (**A**), Scenario 1) is hypothesis-generating: it has not been validated prospectively and is not endorsed by current PIDS/IDSA or ESPID guidance (see Section 4.1.2). The 4-year threshold is an operational simplification modulated by clinical variables (see Section 4.1.1). Gonococcal arthritis in sexually active adolescents requires specific testing (see Section 3.9) and is not included in this matrix. For legibility, reference citations are given in this legend rather than within the figure. CRP = C-reactive protein; ESPID = European Society for Paediatric Infectious Diseases; GA = general anaesthesia; IDSA = Infectious Diseases Society of America; OP = oropharyngeal; PCR = polymerase chain reaction; PIDS = Pediatric Infectious Diseases Society; PVL = Panton–Valentine leukocidin; *speA* = gene encoding streptococcal pyrogenic exotoxin A; TSST-1 = toxic shock syndrome toxin-1.

Together, the advantages of performing arthrocentesis under general anaesthesia in the operating room and the joint-specific evidence on drainage techniques support a risk-stratified approach to bacteriological confirmation in paediatric SA (Figure 2). The appropriate modality of confirmation varies according to the sampling method, the child’s age, the oropharyngeal PCR result, and clinical severity. The proposed framework is detailed in Section 4.1. The choice of approach therefore depends on clinicobiological severity, the pre-test probability of purulent SA, institutional resources and expertise, and the suspected virulence profile [1,2,61,62]. Appendix A compares international guideline recommendations on the indications for joint aspiration.

### 3.4. What Is the Optimal Culture Technique for Joint Fluid?

The diagnostic yield of joint fluid culture is strongly influenced by the method used. Reported positivity rates have historically ranged from 40% to 70%, reflecting both technical variability and differences in patient selection [1,19]. In a prospective study of 40 paediatric patients with OAIs (17 SA, 13 osteomyelitis, and 10 with both), Shin et al. showed that inoculating operative specimens into blood culture bottles (BCBs) improved pathogen detection compared with swab culture (68% vs. 45%) and tissue culture (68% vs. 38%) [63]. Nine samples (23%) were positive only in BCBs, and pathogen identification was faster with BCBs than with the other methods (3.5 vs. 4.3–4.4 days) [63]. These findings support the routine use of BCBs for joint fluid specimens whenever aspiration is performed.

Beyond culture, joint fluid should undergo several complementary analyses. Synovial white blood cell (WBC) count remains an important diagnostic marker: values above 50,000 cells/mm^3^ are classically considered suggestive of bacterial SA, although this threshold is less sensitive in *K. kingae* infections, where counts may be substantially lower [2,26]. Gram stain is highly specific when positive but has only 30–50% sensitivity in paediatric SA, reflecting both the low bacterial burden in many cases and the difficulty of detecting Gram-negative organisms such as *K. kingae* [1,19]. Blood cultures should also be obtained systematically. Overall, they are positive in about 20–50% of paediatric SA cases, with a higher yield in *S. aureus* infections (up to 40–50%) than in younger children, in whom *K. kingae* predominates and blood cultures are almost always negative [1,3,27]. This age-related difference further supports the use of molecular techniques in young children.

Section et al. analysed 4537 cultures from 869 children and provided an important clarification: anaerobic, fungal, and acid-fast bacillus (AFB) cultures should not be performed routinely in haematogenous OAI [19]. These specialised cultures should be reserved for immunocompromised patients, penetrating injuries, or failure of primary treatment [19]. In paediatric SA, the recommended approach is to inoculate joint fluid directly into paediatric blood culture bottles, send an additional specimen for Gram stain and cell count, and perform specific *K. kingae* PCR on joint fluid in children younger than 4 years [19,20,21]. Appendix B (Table A2) summarises the diagnostic yield of the different sampling methods.

### 3.5. Does Antibiotic Pretreatment Compromise Joint Fluid Culture Yield?

A common concern is that giving antibiotics before joint fluid sampling may reduce pathogen detection and therefore justify delaying treatment. Current evidence does not support this view. In a retrospective study of 584 children with OAI, Lansell et al. found no significant difference in culture positivity between those who received antibiotic pretreatment and those who did not, regardless of sampling modality: blood cultures (OR 0.90), bone cultures (OR 0.77), and joint cultures (OR 0.71) [64]. Similarly, the 2021 PIDS/IDSA guideline reported that specimens obtained within 24–48 h after antibiotic initiation had yields comparable to pre-antibiotic samples [1]. Ilharreborde et al. further showed that specific real-time PCR for *K. kingae* remained feasible for up to 6 days after antibiotic initiation [25], extending the diagnostic window when molecular methods are used.

Thus, antibiotic pretreatment does not appear to meaningfully reduce joint fluid culture yield, and antibiotic therapy should therefore not be delayed to obtain cultures. However, most available data come from retrospective studies, and the effect of longer antibiotic exposure (>48 h) on culture sensitivity remains uncertain. When aspiration is indicated, it should be performed as soon as feasible without compromising the priority of prompt antibiotic administration.

### 3.6. Which Clinical and Biological Predictors Support the Need for Invasive Sampling in Children with Suspected SA?

In suspected SA, clinical prediction serves two purposes: distinguishing SA from conditions with similar presentations—such as transient synovitis, reactive arthritis, inflammatory arthropathy, and, more rarely, malignancy—and identifying children most likely to harbour a virulent organism warranting thorough microbiological investigation and treatment.

To address the first question, Kocher et al. proposed a four-variable algorithm—fever, non-weight-bearing, ESR > 40 mm/h, and WBC > 12,000/mm^3^—to differentiate SA from transient synovitis of the hip [65]. The model was later validated in an independent cohort (AUC 0.86) [66]. Caird et al. subsequently added CRP > 20 mg/L as a fifth predictor, increasing the estimated probability of SA to 98% when all five criteria are present [67]. This five-parameter model was later supported by the systematic review by Nannini et al. (11 studies, 391 paediatric hip SA patients), which reported a predictive value of 97.5% [68].

However, the rise of *K. kingae* as the predominant pathogen in young children has substantially reduced the utility of these criteria. The Kocher and Caird criteria were developed and validated before *K. kingae* was widely recognized, and false-negative cases related to this organism have since been reported in multiple centres. This limitation was demonstrated by De Marco et al. in a landmark multicentre retrospective study of 140 children with confirmed *K. kingae* septic arthritis of the hip across 17 hospitals in 7 countries [69]. Of the 77 patients with complete data for all four Kocher variables, only 5 (6.5%) met all four criteria; the median Kocher score was 2 (IQR 1–3), and 63.6% had a predicted probability of SA below 40% despite confirmed infection. Temperature > 38.5 °C and ESR > 40 mm/h were each present in fewer than half of cases, whereas CRP > 20 mg/L was the most sensitive individual predictor (76.2%) [69]. Taken together, these findings show that the Kocher–Caird criteria cannot reliably exclude SA in preschool-aged children when *K. kingae* is the likely pathogen.

A recent meta-analysis by Tang et al. (11 studies, 1810 cases) quantified the discriminative value of individual predictors for differentiating SA from transient synovitis [70]. A history of fever was the strongest predictor (OR 6.04, 95% CI 2.44–14.97, *p* < 0.001), followed by non-weight-bearing status (OR 5.23, *p* = 0.015), ESR (OR 3.98, *p* = 0.017) and WBC count (OR 2.73, *p* = 0.013). CRP alone was not a significant discriminator (OR 7.12, 95% CI 0.59–85.70, *p* = 0.122), challenging its inclusion as an independent predictor [70]. Fever and functional impairment thus remain the most reliable clinical indicators, while laboratory markers should be interpreted in combination rather than in isolation. The non-significance of CRP as an independent discriminator in the Tang meta-analysis (*p* = 0.122) contrasts with its inclusion as the fifth criterion in the Caird model, where CRP > 20 mg/L raises the predicted probability to 98% when all five criteria are present [67]. This discrepancy likely reflects the difference between CRP as a standalone predictor and CRP as a component of a multivariate model, where its contribution is complementary to clinical signs rather than independently discriminative.

Beyond CRP, procalcitonin (PCT) is the most extensively studied biomarker. A meta-analysis of six studies (654 children) reported a pooled sensitivity of 72% and specificity of 90% for PCT in osteoarticular infections, with a positive likelihood ratio of 3.87 but a negative likelihood ratio of only 0.39 [71]. PCT is therefore a moderate rule-in test but cannot reliably exclude infection. The available evidence, moreover, supported the use of PCT more in osteomyelitis than in SA specifically [71].

Direct comparison favours CRP. In a cohort of 54 children with acute monoarthritis, CRP discriminated SA from transient synovitis with high accuracy (AUC 0.950), whereas PCT showed poor discriminative ability (AUC 0.574) and failed to differentiate the two conditions [72]. Two mechanisms explain this: PCT synthesis is driven principally by Gram-negative endotoxin and is poorly stimulated by Gram-positive organisms, and an early localised joint infection may not generate sufficient systemic inflammation to raise PCT [72]. Both are directly relevant to paediatric SA, which is typically localised and, in young children, often accompanied by a mild inflammatory response [26,43]. No study has evaluated PCT specifically in *K. kingae* SA.

Novel biomarker assays have also been explored. In a pilot study of 20 children, a four-marker signature (CTX-II, TGF-α, MCP-1, BCA-1) distinguished SA from osteomyelitis with 90% sensitivity and 80% specificity [73]. However, this assay separated SA from osteomyelitis—not from non-infectious conditions—and included only 10 children with SA; PCT was not retained in the final model.

CRP also plays a central role in aetiological prediction. Ceroni et al. showed that young children with *K. kingae* SA present markedly lower fever rates (<15%) and lower CRP levels—40% have a normal CRP—compared with *S. aureus* infections [26]; Coulin et al. and Basmaci et al. further refined this distinction, consistently identifying older age, higher CRP, and higher fever as independent predictors of *S. aureus* aetiology [43,44]. In the proposed framework, a CRP > 20 mg/L thus serves a dual purpose: it argues against transient synovitis (Section 3.6) and, in children younger than 4 years, predicts a non-*K. kingae*—typically *S. aureus*—aetiology (Section 4.1). These are two distinct clinical questions and should be interpreted accordingly. When aetiological prediction points towards *S. aureus*, escalation towards invasive sampling with culture, antimicrobial susceptibility testing and, where indicated, toxin profiling is warranted [43,44,69]; the severity markers associated with PVL-positive infections (see Section 3.2) should further strengthen this indication.

**Figure 3:**
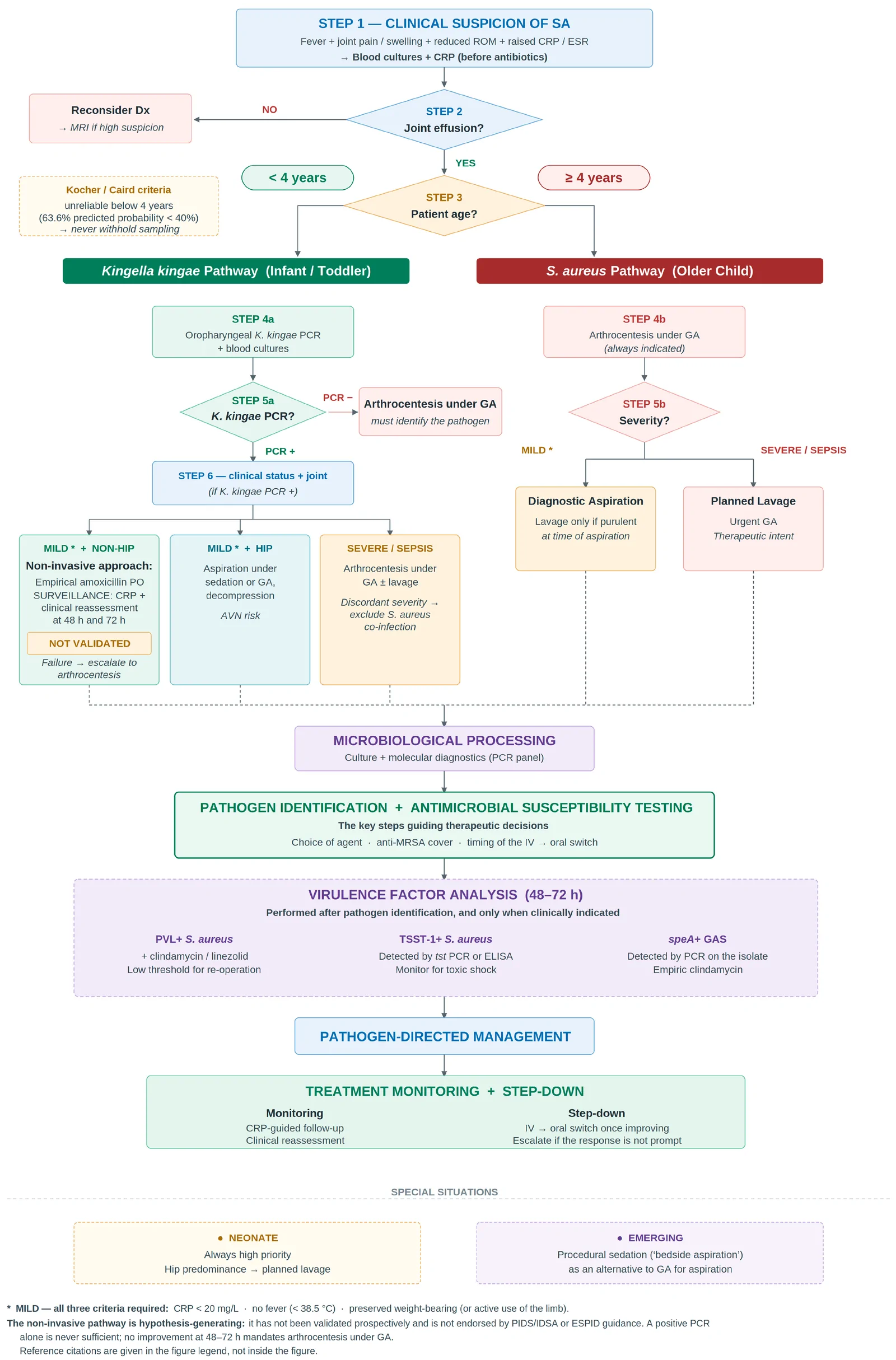
Risk-stratified sampling decision algorithm for paediatric septic arthritis. The algorithm proceeds through six numbered steps. Steps 1–3 apply to every child: clinical suspicion of SA, with blood cultures and CRP obtained before any antibiotic is given [2]; confirmation of a joint effusion; and age-based stratification. In children younger than 4 years (Steps 4a–6), an oropharyngeal *K. kingae* PCR is obtained [31]. A negative result excludes *K. kingae*, makes a more virulent pathogen likely and mandates arthrocentesis under general anaesthesia; a positive result is interpreted together with clinical severity and joint site [26,43,44]. In children aged 4 years or older (Steps 4b–5b), arthrocentesis under general anaesthesia is always indicated, and severity determines whether the procedure is primarily diagnostic or a planned lavage [16,17]. Whatever the pathway, every specimen then enters the routine microbiological sequence: (1) microbiological processing of the sample; (2) pathogen identification and antimicrobial susceptibility testing, which are the key steps guiding therapeutic decisions and the primary microbiological determinants of definitive therapy [1,2]; and only then (3) virulence factor analysis (PVL, TSST-1, *speA*), which is performed after pathogen identification and only when clinically indicated—an adjunctive investigation restricted to severe, multifocal or atypical presentations [16,17,42,58]. Side notes recall that the Kocher–Caird criteria are unreliable below 4 years of age—the median score is 2, and 63.6% of children with confirmed *K. kingae* hip septic arthritis had a predicted probability below 40%—so that they must never be used to withhold sampling [69]; that neonates below 3 months follow a separate pathway, with a different pathogen spectrum and planned lavage from the outset [38,39,40]; and that procedural sedation (‘bedside aspiration’) is an emerging alternative to general anaesthesia, having reduced the time to obtaining joint fluid from 15.7 h to 7.4 h and avoided the operating room in 54% of children [60,61]. The need for empirical anti-MRSA cover depends on local epidemiology, MRSA being rare in Europe (0.7% in the EUCLIDS study) but frequent in North America [1,33,34,35]. The non-invasive pathway is hypothesis-generating: it has not been validated prospectively and is not endorsed by current guidelines (Section 4.1.2). For legibility, reference citations are given in this legend rather than within the figure. CRP = C-reactive protein; ELISA = enzyme-linked immunosorbent assay; ESPID = European Society for Paediatric Infectious Diseases; ESR = erythrocyte sedimentation rate; EUCLIDS = European Union Childhood Life-Threatening Infectious Disease Study; GA = general anaesthesia; IDSA = Infectious Diseases Society of America; MRI = magnetic resonance imaging; (CA-)MRSA = (community-associated) methicillin-resistant *Staphylococcus aureus*; OP = oropharyngeal; PCR = polymerase chain reaction; PIDS = Pediatric Infectious Diseases Society; PVL = Panton–Valentine leukocidin; SA = septic arthritis; *speA* = gene encoding streptococcal pyrogenic exotoxin A; *tst* = gene encoding toxic shock syndrome toxin-1; TSST-1 = toxic shock syndrome toxin-1. Color code: green = K. kingae pathway; red = S. aureus pathway; yellow = decision points; blue = procedural steps.

Taken together, biochemical markers may refine the pre-test probability of SA but cannot identify the pathogen, provide an antibiogram, or detect virulence factors; they complement rather than replace microbiological documentation. CRP remains the single most informative biochemical parameter—used for differential diagnosis, aetiological prediction, and treatment monitoring—whereas PCT adds little as a standalone test in suspected SA. Appendix C summarises these clinical and biological predictors. These age- and severity-based decision points are incorporated into the sampling algorithm shown in Figure 3.

### 3.7. Does Pathogen Identification Change Management in SA?

Section 3.3 established that aspiration has intrinsic therapeutic value. This section asks whether microbiological identification further influences management.

The routine microbiological pathway that follows any positive culture remains the backbone of management. Joint fluid inoculated into paediatric blood culture bottles is incubated and, when growth occurs, the isolate is identified to species level and antimicrobial susceptibility testing is performed [19,63]. Species identification and the resulting antibiogram are the primary microbiological determinants of definitive antimicrobial therapy: they dictate the choice of agent, the need for anti-MRSA cover, and the timing of the intravenous-to-oral switch [1,2]. The characterisation of virulence factors (PVL, TSST-1, *speA*) is, by contrast, a second-line and adjunctive investigation. It is undertaken only after the organism has been identified, is in practice restricted to *S. aureus* and *S. pyogenes*, and is indicated only when the clinical course is severe, multifocal or otherwise atypical [16,17,42]. Where cultures remain negative, molecular methods—specific *K. kingae* PCR, broad-range 16S rRNA PCR, or metagenomic sequencing—are used to close the diagnostic gap (Section 3.8) [20,21,25]. The framework proposed in this review therefore does not displace routine microbiology: it is built upon it.

Culture-negative SA represents 30–60% of paediatric cases, depending on the diagnostic methods used [1,19,25]. Many cases are likely due to fastidious organisms, especially *K. kingae* in young children, which are often missed by conventional culture but detectable by molecular methods [20,25]. Overall, culture-negative SA appears to have a favourable prognosis. In 157 children with SA, Spyridakis et al. reported lower inflammatory markers, shorter hospital stays, and consistently favourable outcomes among culture-negative patients treated empirically [41]. Pääkkönen et al. similarly found that negative cultures did not worsen outcomes in 131 children with OAI [74]. These data support empirical treatment when no pathogen is identified.

When cultures are positive, pathogen identification informs management in three ways. First, it supports antimicrobial stewardship: in 69 children with uncomplicated OAI, Wheeler et al. showed that culture-positive cases could be narrowed to a single targeted antibiotic, limiting unnecessary broad-spectrum therapy [75]. Second, a negative result obtained with appropriate methods, including BCB inoculation and molecular testing, helps rule out virulent organisms and reduces diagnostic uncertainty. Third, in complicated SA due to PVL-positive *S. aureus* or invasive Group A *Streptococcus*, identifying virulence factors directly guides anti-toxin therapy (clindamycin or linezolid), the surgical strategy (repeat lavage versus single-stage management), and the overall intensity of care [16,17,50].

Thus, pathogen identification has a graded clinical impact: it is limited in uncomplicated culture-negative SA, where empirical therapy is usually effective, but it may be decisive in complicated cases by directing both medical and surgical management [16,50,74,75]. The shortcomings of conventional culture, particularly for *K. kingae*, have therefore encouraged the use of molecular tools to close this diagnostic gap (Section 3.8).

### 3.8. What Can Molecular Tools Add?

The limitations of conventional culture are most apparent for fastidious organisms that are difficult to grow, particularly in OAIs caused by *K. kingae*. Ilharreborde et al. applied specific real-time PCR to joint fluid from 89 children with suspected SA and found that only 36 of 89 (40%) had positive conventional cultures. PCR identified *K. kingae* in 24 of 53 culture-negative cases, establishing it as the leading pathogen in 31 of 60 (52%) documented infections [25]. Real-time PCR on blood DNA extracts was negative in all *K. kingae* cases, indicating that this approach is useful on joint fluid but not on blood for this organism [25].

Beyond *K. kingae*-specific PCR, metagenomic next-generation sequencing (mNGS) is an important advance in the molecular evaluation of suspected OAI. It has been shown to identify the causative pathogen in 93% of cases, including several infections missed by conventional culture [76]. Plasma-based mNGS (‘liquid biopsy’) for detecting circulating microbial DNA may further reduce the need for invasive sampling, as illustrated by Ahmed et al. in *K. kingae* spinal infections [77]. However, mNGS remains resource-intensive, with limited standardisation and uncertain cost-effectiveness [78].

Ceroni et al. argued that molecular diagnostics are progressively closing the diagnostic gap left by conventional culture and may signal the end of the culture-negative era [20]. In SA, particularly in children younger than 4 years, the key question is shifting from whether invasive aspiration is needed for culture to which specimen is most appropriate for molecular testing.

### 3.9. Are There Age-Specific Populations Requiring Special Attention in the Sampling Decision?

Two clinical scenarios warrant specific consideration in the context of SA. In neonates, Lee et al. described six cases of hip SA in which initial ultrasound- or fluoroscopy-guided aspiration was negative, with a mean diagnostic delay of 15.2 days [38]. Four patients (67%) had extracapsular abscesses with perforated joint capsules. These findings show that the absence of aspirable fluid does not exclude SA in neonates and that this age group requires a high index of suspicion regardless of aspiration results [38]. In addition, the range of pathogens causing neonatal SA is broader and differs from that seen in older children. Mediamolle et al. (71 neonates younger than 3 months with OAI) confirmed this distinct epidemiology, identifying *Streptococcus agalactiae* as the predominant pathogen (45%), followed by *S. aureus* (22%), while Gram-negative bacilli (*Escherichia coli*, *Klebsiella* spp.) accounted for many cases; late orthopaedic sequelae occurred in 4 of the 34 infants followed for more than one year (11.8%), all after delayed antibiotic initiation (6–12 days) and delayed surgery (6–13 days) [39]. This distinct bacteriological spectrum—including Gram-negative organisms rarely encountered in older children—supports systematic sampling in neonates.

In this population, the hip (32%) and knee (32%) are affected with equal frequency, and surgery is required in 87.3% of cases [39]. A separate systematic review and meta-analysis by Tang et al. (13 studies, 258 neonates) reported a favourable outcome in only 69.7% (95% CI 60.5–77.7%), indicating that about one third of neonates develop adverse sequelae [40]. Longer follow-up (>2 years), non-hip involvement, and prompt intervention within 7 days of symptom onset were identified as significant predictors of a positive outcome [40]. These findings reinforce the notion that neonatal SA is a high-risk condition in which systematic aspiration and surgical exploration are warranted regardless of initial aspiration findings, and that early intervention within the first week is a modifiable prognostic factor [40]. Because these neonatal data differ in study population and outcome definitions [39,40], they should be interpreted as complementary estimates rather than conflicting findings.

Finally, gonococcal arthritis should be included in the differential diagnosis of acute monoarthritis in sexually active adolescents. Because *Neisseria gonorrhoeae* requires specific culture conditions (chocolate agar, Thayer–Martin medium) and nucleic acid amplification testing on joint fluid, it should be suspected in patients presenting with migratory polyarthralgia, tenosynovitis, or dermatitis [2,3]. The 2023 PIDS/IDSA guideline recommends evaluating all sexually active adolescents with acute monoarthritis for gonococcal infection, regardless of initial clinical suspicion [2]. As standard culture protocols may miss this pathogen, specific testing should be requested at the time of joint fluid collection when clinically indicated.

## 4. Synthesis and Clinical Implications

To the best of our knowledge, this is the first narrative review to focus specifically on the indications for bacteriological sampling in paediatric SA as distinct from AHO. It synthesises emerging evidence on virulence factors and their detection, incorporates the concept of an SA-specific diagnostic–therapeutic continuum, and proposes a structured risk-stratified framework.

The evidence reviewed across nine clinical questions supports reframing the diagnostic approach to paediatric SA. Rather than relying on the binary question of whether to sample, the decision should be stratified according to clinical context, age-dependent epidemiology, and suspected pathogen virulence. Our argument is therefore not against sampling itself—its dual diagnostic and therapeutic role usually justifies the procedure—but in favour of a risk-adapted framework that treats pathogen virulence as a key determinant of decision-making.

### 4.1. Proposed Risk-Stratified Framework for Paediatric SA

Based on the evidence reviewed, we propose a conceptual framework operationalised in the decision matrix (Figure 2) and algorithm (Figure 3). The framework rests on a single principle: bacteriological confirmation should be obtained in all cases—either through direct joint sampling or, in young children with a mild presentation, through a concordant set of clinical, biological, and molecular findings—while the sampling modality is tailored to age-related epidemiology, oropharyngeal PCR results, and clinical severity.

**Severely ill child at any age (arthrocentesis + planned lavage):** This group includes immunocompromised children, neonates with SA, children with suspected MRSA or PVL-positive *S. aureus* based on local epidemiology, travel history, or clinical severity (CRP > 160 mg/L, bacteraemia, multifocal disease), those with suspected invasive Group A *Streptococcus* infection, those who fail initial empirical therapy, and any child requiring urgent joint decompression. In these situations, comprehensive sampling, including toxin profiling (PVL, TSST-1), is performed as part of a combined diagnostic–therapeutic procedure [1,2,16,17]. An important exception to the infectious framework concerns children whose clinical or imaging findings suggest an alternative diagnosis, such as malignancy or inflammatory arthropathy; in these cases, aspiration remains indicated to clarify the diagnosis and rule out infection.

***S. aureus* probable (>4 years—arthrocentesis under GA**): Children older than 4 years with suspected SA, in whom *S. aureus* is the most likely pathogen and where antibiogram and toxin profiling are expected to guide management. In this group, arthrocentesis under GA fulfils both diagnostic and therapeutic purposes, with intraoperative lavage if the aspirate is purulent. This category also includes any child in whom identification of *S. aureus* virulence factors (PVL, TSST-1) would be expected to influence management decisions [1,2,16,17].

***K. kingae* confirmed (<4 years, oropharyngeal PCR positive, stable—non-invasive confirmation may suffice):** Children younger than 4 years with clinical and biological features suggestive of *K. kingae* SA and a positive oropharyngeal *K. kingae* PCR may be managed with non-invasive bacteriological confirmation. For the purposes of this framework, a mild clinical presentation is defined by all three of the following objective criteria: (i) CRP < 20 mg/L; (ii) absence of fever (temperature < 38.5 °C); and (iii) preserved ability to bear weight or maintained ambulation (or, for non-weight-bearing joints, preserved active use of the limb). A child who does not fulfil all three criteria falls outside the non-invasive pathway. This approach is supported by the predictable susceptibility profile of *K. kingae* (see Section 3.1), the excellent prognosis in the absence of severity criteria, and the very low yield of conventional culture [26,29,31]. Arthrocentesis is reserved for clinical failure—defined as the absence of a CRP decline or of clinical improvement (persistent fever, worsening joint signs, or failure to resume weight-bearing) within 48–72 h despite empirical antibiotic therapy—diagnostic uncertainty, or the need for therapeutic joint decompression. For children managed non-invasively, a structured surveillance protocol with CRP monitoring at 48–72 h is recommended. A negative oropharyngeal PCR effectively excludes *K. kingae* and should redirect the diagnostic work-up towards other pathogens, thereby strengthening the indication for invasive sampling [31].

No prospective study has validated a management pathway omitting joint aspiration in this population, and this recommendation reflects the authors’ interpretation of the available observational data; it should be applied with close clinical surveillance and a low threshold for escalation to arthrocentesis.

#### 4.1.1. Nuances and Limitations of the Proposed Framework

This selective non-invasive pathway may seem at odds with the diagnostic–therapeutic rationale for aspiration outlined in Section 3.3, where joint decompression is an inherent benefit of arthrocentesis. However, the need for decompression varies by clinical presentation. In young children, *K. kingae* SA usually causes small, serous or seropurulent effusions with limited intra-articular inflammation, unlike the large, frankly purulent effusions more typical of *S. aureus* SA [26,29]. In these low-pressure effusions, inflammation is milder and the proteolytic cartilage damage that warrants urgent decompression in *S. aureus* SA is less likely [79]. The non-invasive pathway should therefore be reserved for mild presentations; any child with a tense effusion, clear joint distension, or no clinical improvement should undergo arthrocentesis regardless of the oropharyngeal PCR result.

A key limitation of the selective non-invasive pathway is the positive predictive value of oropharyngeal PCR. Because *K. kingae* carriage reaches 10–12% among daycare attendees aged 6–48 months [30], a positive result in a child with joint symptoms does not by itself distinguish true *K. kingae* SA from incidental carriage in conditions such as transient synovitis or reactive arthritis. The diagnostic interpretation must therefore rely on a cluster of concordant findings: a positive oropharyngeal *K. kingae* PCR, confirmed joint effusion, mildly elevated or normal inflammatory markers consistent with *K. kingae* infection (CRP typically < 20 mg/L, low-grade or absent fever) [26,44], and age between 6 months and 4 years, when *K. kingae* predominates [24,27]. When these four elements are present, the probability of *K. kingae* SA may be high enough to justify empirical treatment with close surveillance rather than invasive sampling. However, a formal Bayesian estimate cannot be derived from the available literature because the incidence of SA among oropharyngeal carriers—and thus the required prior probability—has not been reported. Conversely, when the clinical picture is discordant, such as CRP > 20 mg/L or fever > 38.5 °C in a young child with a positive oropharyngeal PCR, incidental carriage becomes more likely and the possibility of *S. aureus* or another virulent pathogen should lower the threshold for arthrocentesis.

A less invasive alternative would be bedside aspiration under procedural sedation in young children with suspected *K. kingae* SA, allowing diagnostic sampling without general anaesthesia. This option merits consideration, but several arguments favour the non-invasive approach in this subgroup. Although joint-fluid PCR is highly sensitive [20,21], it identifies the same pathogen as oropharyngeal PCR without providing additional susceptibility data, as *K. kingae* susceptibility is largely predictable [23,29]. In addition, bedside aspiration of deep joints such as the hip requires expertise that is not universally available and carries a small but real risk of contamination or incomplete aspiration [5,60,61]. Its therapeutic value is also limited, given the typically small, low-pressure, low-inflammatory effusions of *K. kingae* SA. Thus, in a young child with a positive oropharyngeal *K. kingae* PCR, mild inflammatory markers, and a non-tense effusion, the non-invasive pathway may avoid a procedure with only marginal diagnostic and therapeutic benefit. This reasoning does not apply to older children, discordant clinical presentations, or joints with clinically significant effusions requiring decompression.

The 4-year cutoff used in this framework is a practical approximation of a gradual epidemiological shift. The transition from *K. kingae* to *S. aureus* predominance is not abrupt: *K. kingae* can occur up to 6 years of age, and *S. aureus* may occur before age 4 [24,27,43]. Coulin et al. and Basmaci et al. identified CRP > 20 mg/L, temperature > 38.5 °C, and older age as independent predictors of *S. aureus* aetiology [43,44]; these variables should therefore complement age in the diagnostic ‘grey zone’ between about 2 and 6 years. In practice, a child younger than 4 years with features suggestive of *S. aureus*—CRP > 20 mg/L, fever > 38.5 °C, or toxic appearance—should follow the invasive pathway (arthrocentesis under GA with culture, antibiogram, and toxin profiling) regardless of the oropharyngeal PCR result, even if it is positive for *K. kingae*. Conversely, a child just over 4 years with a mild presentation and epidemiological features consistent with *K. kingae* (daycare attendance, recent upper respiratory infection) may warrant oropharyngeal PCR before arthrocentesis. The age threshold should therefore be viewed as a decision aid, not an absolute boundary.

A negative oropharyngeal PCR for *K. kingae* in a child younger than 4 years with suspected SA creates a challenging diagnostic scenario. Although such a result effectively excludes *K. kingae* (negative predictive value approaching 100% in the appropriate age group [31]), it does not identify an alternative diagnosis. The main differential is then between SA caused by another pathogen—most often *S. aureus*, streptococcal species, or less commonly *S. pneumoniae*—and non-infectious conditions such as transient synovitis or reactive arthritis. The decision to proceed to arthrocentesis under general anaesthesia should therefore be based on the overall clinical picture, including the Kocher–Caird criteria (despite their limitations in this age group [69]), the inflammatory response (CRP, fever), functional impairment, and ultrasound findings. Children with a negative PCR but marked inflammation and clinical features suggestive of bacterial SA should undergo arthrocentesis, as excluding *K. kingae* increases the likelihood of a more virulent pathogen. By contrast, children with a negative PCR, minimal inflammation, and preserved function may be managed with close surveillance and reassessment, as their presentation is more consistent with transient synovitis.

In all cases, the sampling strategy should be discussed with parents or caregivers as part of shared decision-making. This is especially important for the non-invasive pathway, where deferring arthrocentesis in a child with suspected SA requires a clear explanation of the surveillance plan, criteria for escalation, and expected clinical course. For arthrocentesis under general anaesthesia, parents should likewise be informed of its dual diagnostic and therapeutic purpose and the possibility of intraoperative escalation to surgical lavage. Incorporating parental understanding and preferences into this discussion may improve follow-up adherence and strengthen the therapeutic alliance.

#### 4.1.2. Important Limitation: The Proposed Non-Invasive Pathway Is Not Validated

The non-invasive route for children younger than 4 years of age with a positive oropharyngeal PCR for *K. kingae* and mild clinical features is, at present, a hypothesis generated from observational data. No prospective multicentre study has validated the safety or the efficacy of omitting joint aspiration in this subgroup. Neither the PIDS/IDSA guidelines [1,2] nor the ESPID guidance [3] endorse such an approach; all currently recommend arthrocentesis when SA is suspected. This pathway must therefore not be read as a recommendation for routine practice: it is presented as a hypothesis to be tested, not as a standard of care.

If this pathway is applied, it should be applied only within a structured surveillance protocol, in centres with established expertise in *K. kingae* infection and with immediate access to arthrocentesis, and only after explicit discussion with the family. Mandatory clinical and laboratory reassessment (physical examination and CRP) should be performed at 48–72 h. Monitoring relies on clinical examination and CRP kinetics rather than on repeat oropharyngeal PCR, because *K. kingae* carriage persists in the pharynx for weeks to months irrespective of the response to treatment [30], and a repeat PCR would therefore carry no information about whether the joint infection is resolving. Any child who fails to improve clinically, or whose CRP does not decline, must proceed without delay to arthrocentesis under general anaesthesia. Prospective validation studies are urgently needed before this strategy can be considered for inclusion in clinical guidelines; a suitable study design is proposed in Section 4.5.

### 4.2. Long-Term Sequelae of Paediatric SA and Their Relevance to the Sampling Decision

While the preceding sections focused on pathogen identification and sampling technique, the risk-stratified framework must also account for the consequences of delayed or inadequate management. The severity of SA sequelae provides a strong additional rationale for prompt aspiration, comprehensive sampling, and timely joint lavage. Vidigal et al. (71 hips, 69 children) reported avascular necrosis (AVN) in 28% of paediatric hip SA cases, with severity increasing with age: 12.5% in children under 6 months, 29.1% between 7 months and 5 years, and 35.4% from 6 to 16 years [80]. Early drainage and continuous irrigation within the first 4 days were associated with better outcomes [80]. Agarwal et al. (18 patients, 23 hips) documented long-term sequelae of infantile hip SA classified by the Choi system, with type IV involvement (complete destruction) in 35% of cases [81].

In a retrospective cohort of 77 children with hip, knee, or ankle SA followed for a mean of 8.5 years, Manz et al. reported persistent symptoms in 28% and objective sequelae in 12% [82]. Independent predictors of poor long-term outcome included articular involvement, older age, female sex, need for surgery, persistent fever, and elevated CRP [82]. These findings support the view that SA carries cartilage-related risks that distinguish it from uncomplicated AHO.

The anatomical site is a key determinant of sequelae. Hoswell et al. reported excellent radiological outcomes in all knees but in only 81.3% of hips [83]. Qi et al. similarly suggested that younger age at infection is associated with more severe hip sequelae [84]. Ultimately, sequelae are driven less by infection type alone than by delayed source control. McNeil et al. found that delayed source control predicted orthopaedic complications more strongly than infection type or PVL status [50]. Overall, long-term outcome depends mainly on pathogen virulence, anatomical site—especially the hip—and, above all, timely surgical decompression and appropriate antimicrobial therapy.

For this review, these findings strengthen the case for prompt joint aspiration in suspected SA. The procedure provides diagnostic sampling and therapeutic decompression, allows immediate progression to surgical lavage when the aspirate is purulent, and—when a virulent organism is identified—supports rapid treatment escalation that may help prevent the severe sequelae reported in historical series [23,81,82,85].

### 4.3. Relationship with the Therapeutic Paradigm and the Evolving Role of Molecular Diagnostics

The decision to sample in SA is closely linked to the current therapeutic paradigm for paediatric OAI. In a companion paper [23], we showed that oral-first and early-switch strategies are feasible in a substantial proportion of paediatric OAIs when the causative organism and its resistance profile are known or predictable. In selected cases—particularly young children with suspected *K. kingae* infection and no severity criteria—initial oral antibiotic therapy may even be appropriate. The large Finnish randomised trial conducted by Peltola et al. found that 20 days of antimicrobial therapy was non-inferior to 30 days for acute haematogenous OAI, provided CRP normalised within the first week [86], underscoring the importance of pathogen identification for safe de-escalation. The relationship is bidirectional: sampling enables targeted therapy, while effective empirical treatment may reduce the need for invasive sampling in uncomplicated cases.

In SA, aspiration’s dual diagnostic and therapeutic role reinforces this relationship. As outlined in Section 3.3, the benefits of joint decompression and lavage justify aspiration in most cases—especially when *S. aureus* is suspected or the effusion is clinically significant—even if culture results are unlikely to alter antibiotic management [1,2,5]. Virulence factor testing adds a third purpose: beyond guiding antibiotic selection, sampling may also indicate the need for anti-toxin therapy, repeat lavage, and closer monitoring [16,17,87].

Beyond guiding pathogen-directed therapy, bacteriological sampling also supports antimicrobial stewardship. As shown earlier (see Section 3.7), culture-positive cases can be de-escalated to a single targeted antibiotic [75], reducing unnecessary broad-spectrum use. In the context of rising antimicrobial resistance, this is an additional advantage of systematic sampling beyond individual patient management.

Against its potential harms, joint arthrocentesis has low procedural morbidity. Reported complications—including iatrogenic infection, haemarthrosis, and transient pain—are rare in paediatric series [1,5]. By contrast, delayed or missed recognition of a virulent organism (e.g., PVL-positive *S. aureus*, MRSA, invasive GAS) may result in severe sequelae, including joint destruction, growth disturbance, and, in extreme cases, limb loss or death [50,81,82,85]. This favourable risk–benefit profile supports systematic aspiration in most presentations of paediatric SA, while recognising that in lower-risk scenarios—such as young children with mild inflammatory markers suggestive of *K. kingae*—non-invasive diagnostic strategies may offer comparable diagnostic yield with even less procedural burden [31,32].

The molecular advances discussed in Section 3.8—from specific PCR to metagenomic sequencing—may gradually shift this balance. If plasma-based mNGS (‘liquid biopsy’) is validated in larger paediatric cohorts, it could enable pathogen identification and resistance profiling without invasive tissue sampling, while joint aspiration and surgical lavage would still be needed for therapeutic decompression regardless of the diagnostic method [38,77,78]. In that setting, the main rationale for arthrocentesis would shift from diagnosis to treatment (decompression and lavage). Meanwhile, rapid point-of-care tools such as the synovial leucocyte esterase strip test (100% sensitivity, 83% specificity in a small paediatric cohort) [88] may complement molecular diagnostics by providing immediate bedside guidance, although further validation is required.

### 4.4. Limitations

Several limitations should be acknowledged. As a narrative review, this work did not follow a formal PRISMA-compliant protocol. The evidence base is largely retrospective, and no prospective randomised trial has compared children with SA managed with versus without diagnostic aspiration; such a study would also raise ethical concerns given the therapeutic role of aspiration. The proposed risk-stratified framework, including its three-tier classification, therefore requires prospective multicentre validation before clinical implementation.

The epidemiology reviewed here mainly reflects Western European data, so caution is needed when extrapolating to settings with higher CA-MRSA prevalence, such as North America or North Africa, where pathogen and toxin identification may be more clinically important [35,51]. In particular, the proposed non-invasive pathway for children younger than 4 years assumes that *K. kingae* predominates in this age group; where CA-MRSA is also common in young children, the threshold for invasive sampling should be lower. This review also did not address the role of MRI in guiding the sampling decision, although MRI is increasingly used to confirm the diagnosis and assess adjacent bone involvement.

The positive predictive value of oropharyngeal *K. kingae* PCR in suspected SA has not been formally quantified. As discussed in Section 4.1.1, interpretation currently relies on a cluster of concordant clinical features rather than on a single test result, and no study has provided a Bayesian estimate of post-test probability in this setting. Data on PVL and other virulence factors are also predominantly observational, and the only RCT on adjunctive anti-toxin therapy (CASSETTE) was underpowered [59]. Likewise, the systematic reviews on drainage techniques [89,90] were based on low-quality studies (median MINORS score 4), which limits the strength of joint-specific recommendations. Moreover, much of the evidence on virulence factors and clinical predictors is derived from mixed osteoarticular infection cohorts rather than from isolated septic arthritis, which may limit the direct applicability of these findings to SA specifically. In addition, 12 of the 91 references cited (13.2%) originate from the authors’ institution [6,20,21,23,24,26,27,31,32,33,39,68]. This concentration reflects the fact that our centre has generated a substantial part of the primary literature on *K. kingae* osteoarticular infection, on oropharyngeal PCR, and on the limitations of the Kocher–Caird criteria in this population. Readers should nonetheless bear this concentration in mind when interpreting the emphasis placed on these topics, and the framework proposed here requires independent validation by groups outside our institution. Finally, the Health Protection Agency (HPA) PVL guidance document [91] is a governmental clinical advisory document that has not undergone formal peer review, and its recommendations should be interpreted accordingly.

### 4.5. Future Perspectives

Several research priorities emerge from this review:

1. Validate the non-invasive pathway prospectively. We propose a prospective, multicentre, randomised non-inferiority trial restricted to children aged 6 months to 4 years with suspected SA, a sonographically confirmed non-tense effusion of a joint other than the hip, a positive oropharyngeal *K. kingae* PCR, and a mild presentation as defined in Section 4.1 (CRP < 20 mg/L, no fever, preserved weight-bearing). Participants would be allocated to (a) non-invasive confirmation with empirical antibiotic therapy and protocolised reassessment at 48–72 h, or (b) standard arthrocentesis under general anaesthesia. The primary endpoint would be composite treatment failure at 6 weeks (persistent infection, unplanned surgical drainage, recurrence, or need for antibiotic escalation). Assuming a failure rate of 5% in the invasive arm, a non-inferiority margin of 5 percentage points, a one-sided alpha of 0.025 and 80% power, approximately 300 children per arm (about 600 in total) would be required—a target attainable only through multicentre collaboration; registries such as CORTICES and EUCLIDS could harmonise data across centres. Secondary endpoints should include exposure to general anaesthesia, length of stay, time to weight-bearing, cost, and radiological outcome at 12 months. A separate trial should compare procedural sedation plus selective operative lavage with systematic arthrocentesis under general anaesthesia.
2. Confirm the virulence predictors in Europe. PVL and agr typing require external validation in European cohorts, where CA-MRSA and PVL-positive *S. aureus* remain uncommon [35,51].
3. Test rapid PVL detection at the bedside. Point-of-care ICT (30 min) [57] could enable same-day treatment decisions and deserve prospective evaluation.
4. Develop non-invasive molecular sampling. Plasma-based mNGS may identify the pathogen and its virulence factors without an invasive procedure [38,77,78].
5. Embed sampling in the wider treatment pathway. Sampling decisions should be integrated into broader algorithms linking diagnosis to management [23], moving towards a strategy matched to each child’s risk.

## 5. Conclusions

The dual diagnostic and therapeutic value of joint aspiration justifies bacteriological sampling in SA more strongly than in any other paediatric OAI. Overall, the evidence supports systematic bacteriological confirmation, with the sampling modality—arthrocentesis or non-invasive molecular confirmation—tailored to age-dependent epidemiology, oropharyngeal PCR results, and clinical severity.

Our analysis supports 4 main conclusions.

1. In children older than 4 years, arthrocentesis under general anaesthesia remains the standard. *S. aureus* predominates; joint fluid culture, pathogen identification and antimicrobial susceptibility testing are central to management, with toxin profiling (PVL, TSST-1) as an adjunctive investigation in severe or atypical cases.
2. In children under 4 years with a positive oropharyngeal *K. kingae* PCR and a mild presentation, non-invasive confirmation may suffice. This remains an unvalidated hypothesis, is not endorsed by current guidelines, and should be applied only under structured surveillance. Arthrocentesis is reserved for clinical failure, diagnostic uncertainty, or the need for decompression and joint lavage. The decision rests on a cluster of concordant arguments and never on the PCR alone, given the 10–12% carriage rate; the 4-year threshold should be modulated within the 2–6-year grey zone.
3. A negative oropharyngeal PCR does not establish an alternative diagnosis. It excludes *K. kingae* and thereby raises the likelihood of a more virulent pathogen; it should therefore lower, not raise, the threshold for arthrocentesis.
4. In severely ill children of any age, comprehensive sampling—culture, identification, antimicrobial susceptibility testing and toxin profiling—is performed as part of a combined diagnostic–therapeutic procedure with planned lavage from the outset.

The documented severity of long-term sequelae—avascular necrosis, growth disturbance and persistent functional impairment in up to one third of neonatal cases (Section 4.2)—reinforces the case for prompt aspiration, as timely joint decompression and pathogen-directed therapy may mitigate these disabling outcomes (Section 4.2). The risk-stratified framework that we propose (Figure 2 and Figure 3) integrates these findings into a practical clinical tool, with the decision matrix stratifying the sampling approach by age, oropharyngeal PCR result and clinical severity.

In practice, immediate arthrocentesis under general anaesthesia is required in the following patients: any child older than 4 years with suspected SA; any child with a hip effusion, irrespective of age or PCR result; any child younger than 4 years with a negative oropharyngeal *K. kingae* PCR, or with a presentation discordant with *K. kingae* (CRP > 20 mg/L, fever > 38.5 °C, or toxic appearance); and any child failing to improve within 48–72 h of empirical therapy. Planned joint lavage from the outset is indicated in neonates, in immunocompromised children, and in any severely ill child—defined here on the basis of published severity indicators [12,13,49] as one presenting with sepsis, bacteraemia, CRP > 160 mg/L, multifocal disease, or suspected MRSA; PVL-positive *S. aureus*; or invasive group A *Streptococcus*. The non-invasive pathway may be considered—outside routine practice, under structured surveillance, and ideally within a protocolised or research setting—in a child aged 6 months to 4 years with a positive oropharyngeal *K. kingae* PCR, a confirmed non-tense effusion of a joint other than the hip, CRP < 20 mg/L, no fever and preserved weight-bearing; failure to improve at 48–72 h mandates arthrocentesis.

## Data Availability

No new data were created or analysed in this study. Data sharing is not applicable to this article.

## Abbreviations

The following abbreviations are used in this manuscript:

AFB	Acid-fast bacillus
AHO	Acute haematogenous osteomyelitis
AUC	Area under the curve
AVN	Avascular necrosis
BCB	Blood culture bottle
CA-MRSA	Community-associated methicillin-resistant *Staphylococcus aureus*
CFU	Colony-forming units
CI	Confidence interval
CORTICES	Children’s Orthopaedic Trauma and Infection Consortium for Evidence-based Study
CRP	C-reactive protein
DNA	Deoxyribonucleic acid
ELISA	Enzyme-linked immunosorbent assay
ESPID	European Society for Paediatric Infectious Diseases
ESR	Erythrocyte sedimentation rate
EUCLIDS	European Union Childhood Life-Threatening Infectious Disease Study
GA	General anaesthesia
GAS	Group A *Streptococcus*
GPIP	Groupe de Pathologie Infectieuse Pédiatrique
HPA	Health Protection Agency
ICT	Immunochromatographic test
ICU	Intensive care unit
IDSA	Infectious Diseases Society of America
MINORS	Methodological Index for Non-Randomized Studies
mNGS	Metagenomic next-generation sequencing
MRSA	Methicillin-resistant *Staphylococcus aureus*
MSSA	Methicillin-sensitive *Staphylococcus aureus*
NWB	Non-weight-bearing
OAI	Osteoarticular infection
OR	Operating room/Odds ratio (as specified in context)
PCR	Polymerase chain reaction
PIDS	Pediatric Infectious Diseases Society
PRISMA	Preferred Reporting Items for Systematic Reviews and Meta-Analyses
PVL	Panton–Valentine leukocidin
PVL-SA	PVL-positive *Staphylococcus aureus*
RCT	Randomised controlled trial
RT-PCR	Real-time polymerase chain reaction
SA	Septic arthritis
SIRS	Systemic inflammatory response syndrome
TSST-1	Toxic Shock Syndrome Toxin-1
WBC	White blood cell count

## Appendix A. Guideline Comparison

A comparison of current guideline recommendations on bacteriological sampling in paediatric septic arthritis.

**Table A1:** Summary of guideline recommendations relevant to joint aspiration and bacteriological sampling in paediatric SA.

Aspect	PIDS/IDSA 2021 (AHO) [1]	PIDS/IDSA 2023 (SA) [2]	ESPID 2017 [3]
Blood cultures	Recommended for all suspected AHO (conditional, low quality)	Recommended for all suspected acute bacterial arthritis	Recommended; positive in 20–50% of cases
Joint aspiration	N/A (AHO guidelines)	Recommended for diagnostic and therapeutic purposes (joint decompression)	Recommended; serves dual diagnostic/therapeutic role
*K. kingae* PCR	Not specifically addressed	Recommended when conventional cultures are negative in children <5 years	Mentioned as an emerging diagnostic tool
mNGS/molecular diagnostics	Not addressed	Recommended when conventional cultures are negative; mNGS emerging	Mentioned for *K. kingae* detection; broad molecular methods not addressed
Imaging before aspiration	Not addressed	Ultrasound recommended as initial imaging modality	Not specifically addressed
When sampling can be deferred	Not addressed; notes that culture-negative cases have comparable outcomes	Not addressed	Not addressed
Strength of evidence	Conditional (moderate quality)	Conditional (moderate quality)	Expert consensus and available evidence

## Appendix B. Diagnostic Yield by Sampling Method

The diagnostic yield of bacteriological sampling methods in paediatric septic arthritis.

**Table A2:** Comparison of diagnostic methods for pathogen identification in paediatric SA, with positivity rates, advantages and limitations.

Diagnostic Method	Positivity Rate	Key Advantage	Key Limitation
Blood cultures	20–50%	Non-invasive (venipuncture), widely available	Low yield; negative in most *K. kingae* infections [1,27]
Joint fluid culture (conventional)	40–70%	Direct pathogen identification + susceptibility	Requires invasive procedure; misses *K. kingae* [1,19]
Blood + joint fluid cultures combined	≈55%	+24% incremental yield over blood alone	Still misses fastidious organisms [1]
BCB inoculation of joint fluid	68%	Superior to swab (45%) and tissue culture (38%) [63]	Requires specific technique at time of sampling
Specific *K. kingae* PCR (joint fluid)	>90% sensitivity	Detects *K. kingae* missed by conventional culture; rapid	Requires joint fluid (invasive); specific to *K. kingae* only [20,21,25]
Oropharyngeal PCR for *K. kingae*	87–100% sensitivity	Non-invasive (throat swab); supports diagnosis	Positive in asymptomatic carriers (10–12%); does not confirm joint infection [31,32]
mNGS (joint fluid/tissue)	93% pathogen identification (vs. 45% culture)	Broad pathogen detection; culture-independent [76]	Resource-intensive; limited standardisation; high cost
Plasma mNGS (‘liquid biopsy’)	Under validation	Non-invasive; detects circulating microbial DNA [38,77,78]	Not yet validated for routine paediatric use
Leucocyte esterase strip test	100% sensitivity, 83% specificity	Rapid (≤2 min); inexpensive; point-of-care [88]	Limited paediatric data; does not identify pathogen

## Appendix C. Clinical and Biological Predictors

The clinical and biological predictors guiding the aspiration decision in paediatric septic arthritis.

**Table A3:** Summary of clinical and biological predictors relevant to the sampling decision in paediatric SA, with source populations and implications.

Predictor	Clinical Significance	Implication for Sampling	Source Population
Kocher criteria (fever, NWB, ESR > 40, WBC > 12k)	SA vs. transient synovitis differentiation (AUC 0.86) [65,66]	Guide aspiration decision; validated for hip SA	282 children (Boston)
Caird: +CRP > 20 mg/L	5th criterion raises probability to 98% when all present [67]	Reinforces aspiration indication	Validation cohort
Age < 4 years	*K. kingae* predominance (>80%); low-virulence pathogens [26,29]	Conventional culture low yield; prefer PCR	43 children (Geneva)
CRP >20 mg/L + T > 38.5 °C + age > 4 y	Predictors of *S. aureus* aetiology (vs. *K. kingae*) [43,44]	Culture + susceptibility testing more important	335 children (Geneva + Paris)
Low CRP + young age + no fever	Suggestive of *K. kingae*; culture likely negative [26]	Non-invasive testing preferred (oropharyngeal PCR)	43 + 90 children (Geneva + Paris)
Fever (OR 6.04)	Strongest predictor differentiating SA from transient synovitis [70]	High sensitivity; supports aspiration decision	1810 cases (meta-analysis)
Non-weight-bearing (OR 5.23)	Second strongest predictor [70]	Combined with fever, reinforces aspiration indication	1810 cases (meta-analysis)
63.6% of *K. kingae* hip SA had predicted probability <40% (median Kocher score 2)	Kocher criteria insufficient to exclude SA when *K. kingae* suspected [69]	Lower threshold for aspiration in young children	140 children (17 hospitals, 7 countries)

## Appendix D. Summary of Key Studies

A summary of key studies informing the risk-stratified framework. No randomised controlled trial has addressed the sampling decision in paediatric SA; the evidence base consists predominantly of retrospective cohort studies and case series, with only one RCT (CASSETTE) addressing a related question (adjuvant anti-toxin therapy).

**Table A4:** Summary of key studies informing the risk-stratified framework for bacteriological sampling in paediatric septic arthritis.

Study	Design	*n*	Key Finding	Ref.
Skaggs 2025	Retrospective cohort	62	Aspiration under procedural sedation reduced time to diagnosis (7.4 vs. 15.7 h); 54% avoided OR	[60]
Braig 2022	Multicentre retrospective	233	Aspiration under procedural sedation avoided OR in 49% of cases	[61]
Shin 2020	Prospective	40	BCB culture yield 68% vs. swab 45% vs. tissue 38%	[63]
Lansell 2021	Retrospective	584	No significant difference in culture positivity with antibiotic pretreatment (joint OR 0.71)	[64]
De Marco 2025	Multicentre retrospective	140	Among 77 with complete KC data: median Kocher score 2; 63.6% had predicted probability <40%; only 6.5% met all 4 criteria	[69]
Tang 2024	Meta-analysis	1810	Fever strongest predictor (OR 6.04) for differentiating SA from transient synovitis; CRP not significant alone	[70]
Ilharreborde 2009	Retrospective + PCR	89	PCR identified *K. kingae* in 24/53 culture-negative cases; leading pathogen in 52%	[25]
Donders 2021	Systematic review	279 knees	Arthroscopy: 4% re-intervention vs. 35% arthrocentesis vs. 17% arthrotomy	[89]
Donders 2022	Systematic review	406 hips	Arthrotomy: 3% re-intervention but paradoxically inferior clinical outcomes	[90]
Ceroni 2010	Prospective cohort	43	*K. kingae*: <15% febrile, 40% normal CRP; specific RT-PCR diagnostic standard	[26]
Spyridakis 2019	Retrospective	157	Culture-negative SA: lower inflammatory markers, shorter stay, favourable outcome	[41]
Tang 2026	Systematic review + meta-analysis	258 neonates	Favourable prognosis in 69.7% of neonatal SA; early intervention (≤7 d) is positive predictor	[40]
Campbell 2022 (CASSETTE)	RCT	34	Adjuvant clindamycin: mortality 0% vs. 24% (secondary outcome; NS, underpowered; pilot, mixed adults/children, 11/34 paediatric)	[59]
Hoswell 2019	Retrospective	64	Radiological outcomes: 100% excellent for knee vs. 81.3% for hip	[83]

BCB = blood culture bottle; CRP = C-reactive protein; NS = not statistically significant; OR (in statistics) = odds ratio; OR (in context) = operating room; PCR = polymerase chain reaction; RCT = randomised controlled trial; RT-PCR = real-time polymerase chain reaction; SA = septic arthritis.
